# Self‐Organization of Tissue Growth by Interfacial Mechanical Interactions in Multilayered Systems

**DOI:** 10.1002/advs.202104301

**Published:** 2022-02-09

**Authors:** Tailin Chen, Yan Zhao, Xinbin Zhao, Shukai Li, Jialing Cao, Jun Guo, Wanjuan Bu, Hucheng Zhao, Jing Du, Yanping Cao, Yubo Fan

**Affiliations:** ^1^ Key Laboratory for Biomechanics and Mechanobiology of Ministry of Education Beijing Advanced Innovation Center for Biomedical Engineering School of Biological Science and Medical Engineering School of Engineering Medicine Beihang University Beijing 100083 China; ^2^ State Key Laboratory of Advanced Design and Manufacturing for Vehicle Body College of Mechanical and Vehicle Engineering Hunan University Changsha 410082 China; ^3^ Institute of Biomechanics and Medical Engineering Department of Engineering Mechanics School of Aerospace Engineering Tsinghua University Beijing 100084 China

**Keywords:** biomechanics, compression gradient, interfacial interaction, morphogenesis, self‐organization, tissue fluidity

## Abstract

Morphogenesis is a spatially and temporally regulated process involved in various physiological and pathological transformations. In addition to the associated biochemical factors, the physical regulation of morphogenesis has attracted increasing attention. However, the driving force of morphogenesis initiation remains elusive. Here, it is shown that during the growth of multilayered tissues, a morphogenetic process can be self‐organized by the progression of compression gradient stemmed from the interfacial mechanical interactions between layers. In tissues with low fluidity, the compression gradient is progressively strengthened during growth and induces stratification by triggering symmetric‐to‐asymmetric cell division reorientation at the critical tissue size. In tissues with high fluidity, compression gradient is dynamic and induces cell rearrangement leading to 2D in‐plane morphogenesis instead of 3D deformation. Morphogenesis can be tuned by manipulating tissue fluidity, cell adhesion forces, and mechanical properties to influence the progression of compression gradient during the development of cultured cell sheets and chicken embryos. Together, the dynamics of compression gradient arising from interfacial mechanical interaction provides a conserved mechanism underlying morphogenesis initiation and size control during tissue growth.

## Graphical abstract

Multilayered tissues can be self‐organized by the progression of a compression gradient stemmed from the interfacial shear stress between layers. Tissue fluidity has profound effects on this process. In solid‐like tissues, the compression gradient is progressively strengthened and induces stratification by triggering asymmetric cell division. In fluid‐like tissues, the compression gradient is dynamic and induces cell rearrangement leading to 2D in‐plane morphogenesis.

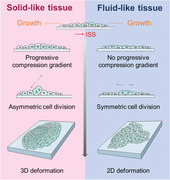

## 1 Introduction

Morphogenesis is a common process that occurs widely in embryonic development, tissue regeneration, and cancer progression. The orchestration of morphogenetic processes is complex, and involves spatial and temporal regulation by biochemical factors (e.g., cell polarity signals and morphogen gradient) and physical factors.^[^ ^1^ ^]^ Emerging studies have revealed that proper morphogenesis relies on the mechanical force of the cells and their environment. For example, apical constriction of cells caused by the contractility of myosin,^[^ ^2^ ^]^ cell rearrangement (e.g., cell intercalation),^[^ ^3^ ^]^ and tissue‐stiffness‐dependent cell migration^[^ ^4^ ^]^ has been reported as an important mechanisms in tissue shaping during embryonic development. These studies indicate the essential functions of cellular and molecular mechanics in the progression of morphogenesis. However, the upstream events, especially the initial driving forces of morphogenesis, remain unknown.

Most biological tissues have multilayered structures. The interactions between layers are essential in tissue homeostasis maintenance and morphogenesis during embryonic development and pathological progression.^[^ ^5^ ^]^ For example, the interaction between cancer cells and their adjacent stroma plays a key role in the progression of the diseases, including tumor invasion.^[^ ^6^ ^]^ In addition to biochemical communications, increasing evidence has shown the essential role of physical interactions between adjacent layers in the regulation of morphogenesis.^[^ ^4^, ^7^ ^]^ For instance, follicle formation in chicken embryos is initiated by mechanical forces transduced from the dermal layer to the epidermal layer.^[^ ^8^ ^]^ The villi of human and chicken guts are formed by the compressive stresses generated by smooth muscle layers on the endoderm and mesenchyme layers.^[^ ^9^ ^]^ These studies reveal that morphogenesis processes are dependent on the mutual collaboration and mechanical compatibility of multiple layers. In this sense, it is necessary and important to address the general mechanism underlying the initiation of morphogenesis during the growth of various multilayered tissues.

Here, by combining biological experiments, theoretical analysis, and numerical simulations, we report a general mechanism underlying the initiation of morphogenesis driven by the progression of compression gradient stemmed from the interfacial mechanical interactions between growing tissue layers.

## 2 Results

### 2.1 Progressive Compression Gradient Is Strengthened in Epidermal Layer During Chicken Feather Follicle Morphogenesis

During the development of avian skin, the feather follicles develop by the stratification of single‐layered epidermis.^[^ ^10^ ^]^ In the ex vivo culture of chicken skins, we found that the feather primordia emerged on day 2 in cultured epidermis combined with dermis after isolation from HH30 stage embryos (Figure S1a–c, Supporting Information). However, when epidermal cell sheet was isolated and cultured alone (without dermal cell layer), it failed to stratify, and no primordium was formed (Figure S1b,c, Supporting Information), indicating that the interaction between epidermal and dermal layers is essential for the morphogenetic process of an epidermal cell sheet. Moreover, during the evolution of epidermis from monolayer to multilayer, the shapes of epidermal cells showed significant alteration from flat to columnar and correlated with the stages in embryo development and with the increased epidermal cell layer number (Figure S1d,e, Supporting Information). The deformation degree of epidermal cells was gradually declined with the increased distance to the primordium center (Figure S1f,g, Supporting Information). These experimental observations indicate that, in the beginning of follicle morphogenesis, a local compression gradient was progressively strengthened in the epidermal cell sheet accompanied with the 3D deformation of epidermis.

According to our previous studies about the surface‐wrinkling pattern formation in a nonliving chemical film/substrate composite soft material, the progression of compression gradient could be generated by mismatch deformation between adjacent layers through interfacial mechanical interactions, which leads to intriguing morphogenesis^[^ ^11^ ^]^ (Figure S2, Supporting Information). Indeed, it has been reported that during the development of chicken skins, dermal cells form aggregations and compress the adjacent epidermal cells to form follicle primordium.^[^ ^8^, ^12^ ^]^ Thus, we hypothesize that the mismatch deformation between epidermal and dermal layers by dermal cell aggregation generates a compression gradient through interfacial mechanical interactions and causes stratification of epidermis. However, whether tissue stratification could be triggered by the mechanical interactions between adjacent layers during tissue growth remains elusive.

### 2.2 2D‐to‐3D Morphogenesis Could Be Self‐Organized at a Critical Size in Multilayered Tissues

**Figure 1:**
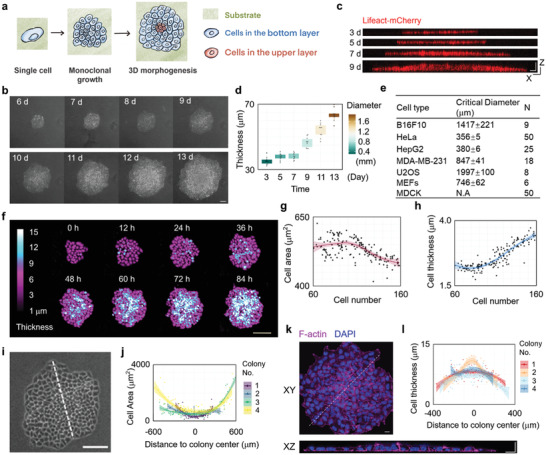
Emergent 3D morphogenesis in a growing monoclonal cell sheet at critical size. a) The illustration of experimental workflow for studying the emergent 3D morphogenesis in a freely growing monolayer HeLa cell sheet. Substrate refers to the ECM attach to the cell culture dish. b) Representative phase contrast images of a growing monoclonal HeLa cell sheet captured at the indicated time (d: day) after seeding. Scale bar: 100 µm. c,d) *XZ* slice images of F‐actin indicated by Lifeact‐mCherry in a growing monoclonal Lifeact‐mCherry^+^ HeLa cell sheet captured at the indicated time after seeding. Scale bar: 50 µm. The statistical analysis of cell sheet thickness and diameter of a growing HeLa cell sheet (*n* = 10). Data are presented as median ± min/max. e) The critical size for 3D morphogenesis of growing cell sheet in different cell types. f) The representative live images of a growing HeLa cell sheet using HoloMonitor M4 time‐lapse cytometer. Scale bar: 150 µm. g) The statistical analysis of the area of individual cell during HeLa cell sheet growth. h) The statistical analysis of the thickness of individual cell during HeLa cell sheet growth. i) The magnified view of HeLa cell sheet on 8 day in panel (b). Scale bar: 100 µm. j) The statistical analysis of the individual cell area along the lines in panel (i). k) Representative *XY* and *XZ* slice images of F‐actin and nucleus stained by phalloidin and DAPI, respectively, in HeLa cell sheet. Scale bar: 20 µm. l) The statistical analysis of the individual cell thickness along the lines in panel (i).

To study the role of interfacial mechanical interactions between layers in the initiation of tissue morphogenesis, we developed a simple film/substrate system composed of a freely growing (without contacting with the boundary) monoclonal cell sheet and extracellular matrix (ECM) (**Figure** **1**a). To ensure the occurrence of stratification, cells without contact inhibitory (which causes decrease in both locomotion and proliferation rates when cell density reaches a certain level^[^ ^13^ ^]^) properties were chosen for this model using a skin‐derived cell line B16F10. During the continuous live imaging, the emergence of a 2D monolayer‐to‐3D multilayer transition was stably observed at a constant critical size (1479 mm diameter) of cell sheet (Figure S3, Supporting Information). Interestingly, similar 2D monolayer‐to‐3D multilayer transition during cell sheet growth was universally observed in different cell types including HeLa, HepG2, MDA‐MB‐231, U2OS, and mouse embryonic fibroblasts (MEFs), except Madin–Darby canine kidney (MDCK) cells, which never showed stratification during our observation (Figure 1a–e). The critical size for 3D morphogenesis during cell sheet growth was relatively constant for a given cell type, indicating a self‐organized mechanism. Among these cell lines, HeLa cells displayed the strongest stratification ability indicated by the smallest critical size (Figure 1e). Thus, we used HeLa cells for the subsequent study. In addition, a similar phenomenon was observed in different types of ECM as well as altered substrate stiffness (Table S1, Supporting Information).

The behaviors of individual cells during cell sheet growth prior to the 3D morphogenesis transition were examined using a holographic imaging cytometer. Although the cell proliferation rate was negligibly altered during cell sheet expansion (Figure S4a, Supporting Information), the average area of individual cells significantly decreased, and the average cell thickness concurrently increased during cell sheet growth, indicating significant cell deformation (Figure 1f–h). Moreover, the largest cell deformation was observed at the central region of the cell sheet (Figure 1i–l). Single cell tracing of cell deformation also suggested significant compression of cells in the central region of cell sheet (Figure S5, Supporting Information). This cell deformation behavior indicates that during cell sheet growth, a compression gradient within the cell sheet emerges.

To further verify the compression gradient, a scratching experiment was performed crossing the center to the edge of the cell sheet. After scratching, cells in the central region showed much faster expansion and migration speed compared with peripheral cells, indicating the release of compressive strain (Figure S6, Supporting Information). This compression gradient was also indicated by traction force microscopy (TFM) analysis and monolayer stress microscopy (MSM) results in a previous study (figure 1 of Perez‐Gonzalez's study).^[^ ^14^ ^]^

**Figure 2:**
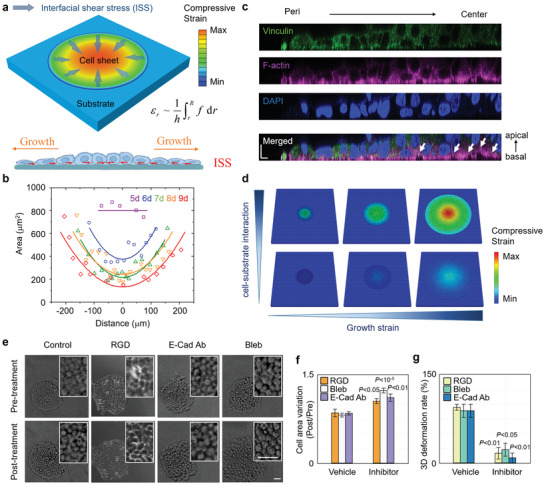
Size‐dependent 3D morphogenesis is induced by interfacial shear stress in multilayered system. a) Theoretical model of a cell monolayer sheet growing on a substrate. *f* is the interfacial shear stress between the cell sheet and substrate generated by relative motion. *h* is the thickness of cell sheet. *σ*_r_ is the compressive stress in the cell sheet induced by the interfacial shear stress. *R* is the outer radius of the cell sheet and *r* is the distance between cell and center of the monolayer sheet. Diagrams of interfacial shear stress between cell and substrate. Direction of interfacial shear stress is contrary to the cell sheet expansion, as the yellow arrow points out. b) Area of the individual cell along a random line across the center of the cell sheet. Data points refer to the experimental results and corresponding lines are theoretical predictions obtained by fitting the experimental data using Equation (S6) (Supporting Information). c) Representative *XZ* slice image of vinculin, F‐actin, and nucleus stained by vinculin antibody, phalloidin, and DAPI, respectively, in half of a HeLa cell sheet. White arrows indicate the cells that were delimitated from the substrate. Scale bar: 10 µm. d) Finite element simulation of the stress field in the growing cell monolayer with different cell–substrate interactions (*f*_top_/*f*_bottom_ = 5). Growth strains from left to right are 10%, 60%, and 120%, respectively. Color bar shows the distribution of the compressive stress in the cell monolayer. e) Representative phase contrast images of HeLa cell sheet in the presence of inhibitors or vehicle. Scale bar: 50 µm. f) The statistical analysis of the individual cell area of HeLa cell sheet in the presence of inhibitors or vehicle (*n* = 10). g) The morphogenesis percentage of HeLa cell sheet in the presence of inhibitors or vehicle (*n* = 3). f,g) Data are presented as mean ± SEM. Each inhibitor's group was compared only with the corresponding vehicle's group by two‐tailed independent‐samples *t*‐test.

To investigate the generation mechanism of compression gradient in the cell sheet during growth, we performed theoretical analysis. As illustrated in **Figure** **2**a, the cell sheet is considered as continuum material. During cell sheet expansion caused by cell proliferation, interfacial shear stress (ISS) would be generated between the cell sheet and substrate layers. Since the direction of ISS is contrary to the relative motion between the adjacent layers, the cell sheet is subjected to ISS directed toward the center, which is consistent with the observations by TFM.^[^ ^15^ ^]^ Thus, cells in the central region of the cell sheet would sustain higher level of compression than those in other regions (Figure 2a; “Mechanical Modeling” section in the Supporting Information), which is confirmed by the experimental observations (Figure 1i–l). The elastic strain energy stored in the cell would increase with the expansion of cell sheet. When the elastic strain energy in the cell is small, interfacial normal adhesion would impose restriction on delamination, making the cell monolayer grow in plane. Thus, higher compressive strain would be generated further. When the compressive strain reaches a critical value, elastic strain energy stored in the cell may be greater than the energy of interfacial normal adhesion and led to the occurrence of interfacial delamination. In this critical condition, stratification may happen, and the elastic strain energy can be released (“Mechanical Modeling” section in the Supporting Information). Indeed, we found that when cell sheet grew beyond the critical size, amounts of cells in the central region were delaminated from the substrate (Figure 2b).

Theoretical analysis can also give valid quantitative predictions of experiments. First, theoretical results of the distribution of cell areas agree well with the experimental results during cell sheet expansion (Figure 2b). According to the theoretical analysis, the critical size of the cell sheet for 3D morphogenesis depends on the mechanical properties of cell sheet and substrate and interactions between them. This is confirmed by finite element simulations, which well resembled the morphogenesis at the cell sheet center and also indicated that, when the compressive strain exceeded the critical value, alterations in the cell–substrate interactions could significantly affect the compression gradient during cell sheet growth (Figure 2d; “Mechanical Modeling” section in the Supporting Information). Moreover, experimentally manipulating cell–substrate interactions using the Arg–Gly–Asp (RGD) peptide, whose soluble form competitively inhibits the binding between a cell and the RGD sequence of a substrate, RGD treatment significantly attenuated the compression gradient and morphogenesis (Figure 2e–g). In addition, inhibition of the contractility of the cell sheet using the cytoskeleton inhibitor blebbistatin or myosin short hairpin RNA (shRNA) significantly disrupted the compression gradient and morphogenesis (Figure 2e–g; Figure S7, Supporting Information). Based on the theoretical analysis, adhesion between cells may also affect the compression gradient. Consistently, inhibition of the cell–cell adhesion force by an E‐cadherin neutralizing antibody also reduced the maximum compressive strain in the cell monolayer and inhibited the morphogenetic process (Figure 2e–g). These results show that the emergent 2D‐to‐3D growth transition of a cell sheet/substrate system could be physically triggered by interfacial mechanical interactions between adjacent layers during growth.

### 2.3 Critical Compression Triggers Symmetric‐to‐Asymmetric Reorientation of Cell Division

**Figure 3:**
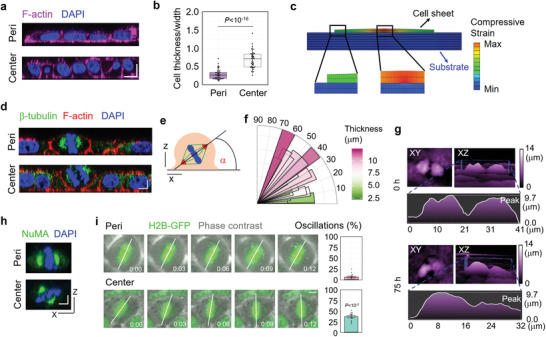
Critical compression triggers cell division reorientation to induce tissue stratification. a) Representative *XZ* slice image of F‐actin and nucleus stained by phalloidin and DAPI, respectively, in the central region and peripheral region of HeLa cell sheet. Scale bar: 10 µm. b) The statistical analysis of cell deformation (thickness/width) in the center region and peripheral region of HeLa cell sheet (*n* = 50). c) Cell shape variation induced by interfacial shear stress. d) Representative *XZ* slice image of *β*‐tubulin, F‐actin, and nucleus stained by *β*‐tubulin antibody, phalloidin, and DAPI, respectively, in the central region and peripheral region of HeLa cell sheet. Scale bar: 5 µm. e) The schematic experimental setting of the mitotic spindle orientation. f) Distribution of the spindle‐axis angles of cells with different thickness in HeLa cell sheet. g) The representative images of dividing cells before (0 h) and after (75 h) critical compression during HeLa cell sheet growth using HoloMonitor M4 time‐lapse cytometer. h) Representative *XZ* slice image of NuMA and nucleus stained by NuMA antibody and DAPI, respectively, in the central region and peripheral region of HeLa cell sheet. Scale bar: 5 µm. i) Analysis of spindle oscillation in the central region and peripheral region of GFP‐H2B^+^ U2OS cell sheet. The extent of oscillation was calculated and plotted in bar graphs on the right. Scale bar: 5 µm (*n* = 14). b) Data are presented as median ± min/max. i) Data are presented as mean ± SEM. b,i) Two‐tailed independent‐samples *t*‐test.

We proceeded to investigate the biological mechanism of the cell layer number increase in the central region of the cell sheet induced by cell layer/substrate interfacial interaction. We found that, in contrast to symmetric (parallel) cell division in the peripheral region of large cell colonies, abundant asymmetric (oblique or perpendicular) cell division was observed in the central cells, which bore significant compression (**Figure** **3**a–f). The orientation of the division plane was closely correlated with cell thickness (Figure 3f). Moreover, after release of compression by cell scratching, the reorientation of cell division was diminished (Figure S8, Supporting Information), indicating the effect of the compression gradient on cell division orientation.

This asymmetric cell division ultimately led at least one daughter cell to locate at the top layer of the cell sheet (Figure 3g; Movies S1 and S2, Supporting Information). The cell division reorientation induced by the compression gradient was also confirmed by the subcellular localization of the nuclear‐mitotic apparatus protein (NuMA), which is involved in the orchestration of mitotic spindle positioning^[^ ^16^ ^]^ (Figure 3h). Moreover, spindle‐rocking experiments indicate that cells in the central region showed a significantly higher level of metaphase plate oscillations, which are always associated with asymmetric cell division^[^ ^17^ ^]^ (Figure 3i). These results suggest that the compression gradient induced by interfacial interaction between layers triggers cell division reorientation from symmetric to asymmetric leading to tissue stratification.

### 2.4 Tissue Fluidity Controls 2D‐to‐3D Morphogenetic Transition by Regulating Compression Gradient Progression

Recall that MDCK cells did not display stratification during our observations (Figure 1e). To further investigate the intrinsic properties of tissue layers that affect the emergent morphogenesis, we compared cell behaviors during cell sheet growth between HeLa and MDCK cells. First, in order to exclude the influence of contact inhibition on MDCK cell behavior, we measured cell proliferation rate and cell density alterations during cell sheet growth. As shown in Figure S4b,d (Supporting Information), MDCK cells did not show contact inhibition during our observation. Moreover, according to the previous report that MDCK cell sheet did not display contact inhibition until a critical size of ≈2 × 10^6^ µm^2^.^[^ ^18^ ^]^ In our experiment, the cell sheet size of MDCK colony was below 1 × 10^5^ µm^2^. Thus, the differential behavior of MDCK cell sheet was not caused by contact inhibition.

**Figure 4:**
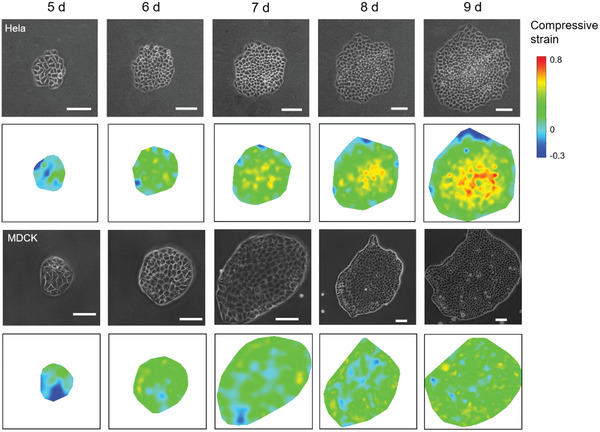
Strain field evolution of HeLa and MDCK cell sheets. The calculated strain field evolution during the growth of HeLa and MDCK cell sheets at the indicated time (d: day) after seeding. Compressive strain field was calculated using Equation (S4) (Supporting Information) by measuring the areas of each cell. The normal area of a full‐grown cell without sustaining compression was set as the average area of the cell sheet in 1 day since the compressive gradient is small in the initial stage. A compression strain greater than 0 indicates compression, and a strain less than 0 indicates tension. Scale bar: 100 µm.

**Figure 5:**
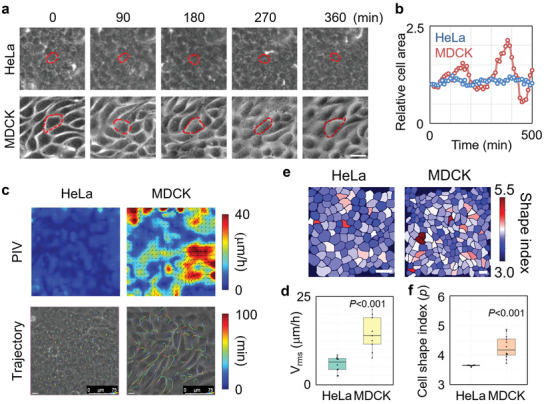
HeLa and MDCK cell sheets show different fluidity during growth. a) The fluctuation of individual cell shape during the growth of HeLa and MDCK cell sheets. Scale bar: 25 µm. b) The area alteration with time of the cell indicated by dotted line in panel (a). c) Cell velocity field analyzed by PIV and cell trajectories in HeLa and MDCK cell sheets. Scale bar: 75 µm. d) The statistical analysis of cell speed (rms velocity) measured by PIV in HeLa and MDCK cell sheets (*n* = 10). e) The cell shape index distribution of HeLa and MDCK cell sheets. Scale bar: 50 µm. f) The statistical analysis of cell shape index of HeLa and MDCK cell sheets (*n* = 10). d,f) Data are presented as median ± min/max; two‐tailed independent samples’ *t*‐test.

Interestingly, we found that, instead of 3D morphogenesis, MDCK cell sheet showed dramatic alterations in its in‐plane shape during growth, with dynamically organized protrusions at cell sheet edges, indicating an emergent 2D morphogenesis (Figure S9, Supporting Information). While a few differences in the proliferation rate were observed between these two cell types (Figure S4a,b, Supporting Information), the expansion rate was significantly higher in MDCK cell sheet than in HeLa cell sheet during growth (Figure S4c,d, Supporting Information). Importantly, in contrast with the HeLa cell sheet behavior, the MDCK cell sheet did not show progressive compression gradient strengthen during growth (**Figure** **4**). Moreover, while the individual cell area in the HeLa cell sheet was relatively steady, MDCK cells exhibited strong area fluctuation during cell sheet growth (**Figure** **5**a,b).

Particle imaging velocimetry (PIV) analysis showed that during cell sheet expansion, MDCK cells displayed rapid collective cellular motion with a higher overall cell velocity (root‐mean‐square (rms) *v*elocity, *v*_rms_), indicating a fluid‐like state.^[^ ^19^ ^]^ In contrast, the collective behavior of HeLa cells showed slower cell motion, indicating a relatively solid‐like state (Figure 5c,d; Movie S3, Supporting Information). The fluidity of these two cell types was further confirmed by the cell shape index *p*_0_, the median ratio of the perimeter to the square root area of the cells. According to the vertex model, if the cell shape index of the system increases to p*_0_ ≈ 3.81, a transition from a jammed, solid‐like state to an unjammed, fluid‐like state occurs.^[^ ^20^ ^]^ Over the course of cell sheet growth, the average cell shape index of MDCK cells was constantly above 3.81, whereas HeLa cells approached the jamming threshold p*_0_ (Figure 5e,f). Moreover, the cell shape index was lower in the central region than in the peripheral region of the HeLa cell sheet (Figure S10, Supporting Information).

**Figure 6:**
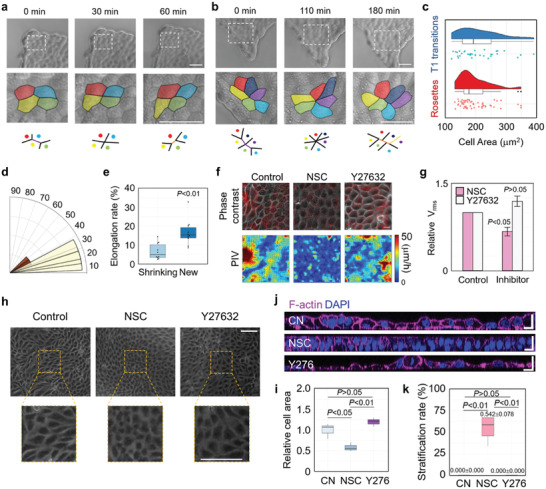
Tissue fluidity controls 2D/3D morphogenetic transition by regulating compression gradient progression. a) Representative image of *T*1 transition at MDCK cell sheet edges during growth. Cell rearrangements are illustrated at the bottom layer (purple line: shrinking junction, orange line: new junction). (min: minute). Scale bar: 50 µm. b) Representative image of rosettes at MDCK cell sheet edges during growth. Cell rearrangements are illustrated at the bottom layer (purple line: shrinking junction, orange line: new junction). Scale bar: 50 µm. c) Cell area distributions of cells will be experiencing *T*1 transitions and rosettes. d) Distribution of the angles between new junctions and tissue protrusion directions. e) Cell sheet elongation rate at the direction of shrinking junctions or new junctions (*n* = 10). f) The velocity field superimposed on the corresponding phase contrast (upper panel) and velocity map (lower panel) images measured by PIV of MDCK cell sheet in the presence of inhibitors or vehicle. Scale bar: 20 µm. g) The statistical analysis of cell speed (rms velocity) measured by PIV of HeLa and MDCK cell sheets in the presence of inhibitors or vehicle (*n* = 4). h) Representative phase contrast images of MDCK cell sheet in the presence of inhibitors or vehicle. Scale bar: 100 µm. i) The statistical analysis of the individual cell area of MDCK cell sheet in the presence of inhibitors or vehicle (*n* = 3). j) Representative *XZ* section images of MDCK cell sheet in the presence of inhibitors or vehicle. Scale bar: 25 µm. k) The stratification percentage of MDCK cell sheet in the presence of inhibitors or vehicle. Stratification percentage values (mean ± SEM) are indicated above the graph (*n* = 4). e,i,k) Data are presented as median ± min/max. g) Data are presented as mean ± SEM. e) Two‐tailed independent‐samples t test. g) Two‐tailed one‐sample t test. i,k) One‐way ANOVA with Tukey's correction.

At the edges of MDCK cell sheet, especially during the formation of protrusions, significant collective cell migration (Movie S4, Supporting Information) and abundant cell intercalations such as *T*1 transitions and rosettes formation were observed (**Figure** **6**a,b). Cell intercalation is reported as a mechanism for driving tissue extension in embryonic development^[^ ^21^ ^]^ and could be controlled by external constraints acting on the tissue.^[^ ^22^ ^]^ In our experiment, most of cell intercalation was observed in cells with relatively smaller areas (less than 300 µm^2^), indicating a high level of compressive strain (Figure 6c). Moreover, the cell rearrangement during the intercalation process was closely correlated with the 2D deformation of cell sheet, indicated by the small angles between new junctions and tissue protrusion directions (Figure 6d) and higher tissue elongation rate at the direction of new junctions (Figure 6e).

To investigate the effect of the tissue fluidity on the 2D/3D morphogenetic transition, cellular migration ability was inhibited using the small GTPase Rac1 inhibitor NSC23766.^[^ ^23^ ^]^ The results showed that Rac1 inhibitor treatment significantly reduced tissue fluidity (Figure 6f,g; Movie S5, Supporting Information) and induced higher level of compressive strain in MDCK cell sheet (Figure 6h,i). Importantly, treatment of NSC23766 significantly promoted the emergence of tissue stratification (Figure 6j,k). In comparison, inhibition of Rho‐associated, coiled‐coil containing protein kinase (ROCK) by Y27632 slightly enhanced tissue fluidity of MDCK cells (Figure 6f,g; Movie S5, Supporting Information) and had little effect on morphogenesis (Figure 6h–k). On the contrary, in HeLa cell sheet, increasing tissue fluidity by Y27632 significantly inhibited the stratification rate (Figure S11, Supporting Information). These results suggest that the morphogenesis driven by interfacial mechanical interactions between tissue layers is dependent on tissue fluidity that higher level of tissue fluidity may prevent the storage of the compressive strain energy through in‐plane cell motion.

### 2.5 Progressive Compression Gradient Contributes to Epidermal Cell Stratification During Chicken Skin Development

Asymmetric cell division is a common mechanism involved in tissue stratification and cell fate differentiation during embryogenesis and cancer progression.^[^ ^24^ ^]^ There is evidence that during the development of chicken skins, the deformation of basement membrane between epidermis and dermis by the aggregation of dermal cells plays a crucial role for the initiation of follicle primordium formation.^[^ ^8^, ^12^ ^]^ According to our results, tissue stratification could be initiated by the mechanical interaction at interface between adjacent layers through compression‐induced cell division reorientation. Thus, we proceeded to verify whether this mechanism is applied to the stratification of epidermis during chicken feather follicle morphogenesis.

**Figure 7:**
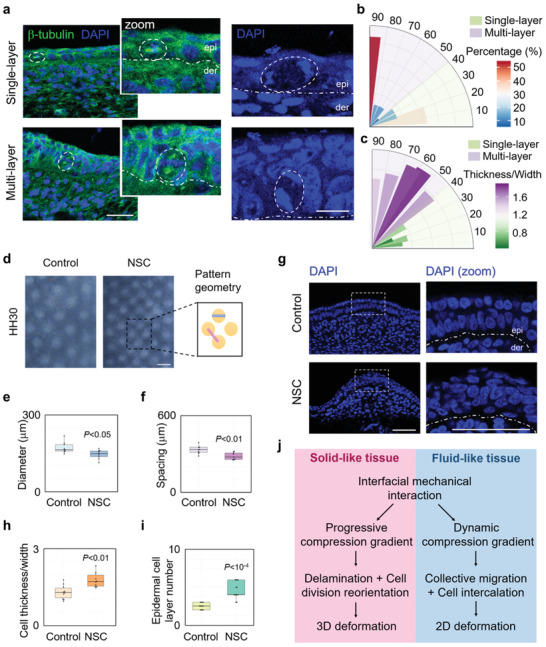
Progression of compression gradient contributes to epidermal cell stratification during chicken skin development. a) Images of embryonic chicken skin showing the orientation of mitoses relative to the basement membrane (white dotted line) in the magnified images (zoom), separating epidermis (epi) from dermis (der). White dotted circles indicate the mitotic cells in metaphase (left) and anaphase (right). DAPI marks the DNA and *β*‐tubulin antibody immunofluorescence staining marks the spindle. Scale bar: 10 µm. b) Distribution of the spindle‐axis angles of cells in single‐layered or multilayered epithelia of embryonic chicken skin. c) Distribution of the spindle‐axis angles of cells with different cell deformation (thickness/width) in single‐layered or multilayered epithelia of embryonic chicken skin. d) Reconstitution culture of embryonic chicken skin with or without NSC23766 (NSC). Scale bar: 500 µm. Quantification of e) spacing and f) diameter as pattern geometry parameters as illustrated in the right panel of panel (d) (*n* = 10). g) Representative cross‐sectional image of nucleus stained by DAPI in reconstitution cultured embryonic chicken skin with or without NSC. Scale bar: 50 µm. h) Quantification of cell deformation of epidermis in the presence of NSC or vehicle (*n* = 10). i) Quantification of layer number of epidermis in panel (g) (*n* = 14). j) Models of 2D and 3D morphogenesis controlled by the interfacial mechanical interactions in multilayered systems with different tissue fluidity. e,f,h,i) Data are presented as median ± min/max, two‐tailed independent samples’ *t*‐test.

First, we compared the cell division orientation of the epidermis in single‐layered stage and multilayered stage during skin development. We found that when epidermis was stratified, most of the mitotic spindles of epidermal cells were reoriented from symmetric to asymmetric, and the spindle‐axis angle was closely correlated with cell deformation degree (**Figure** **7**a–c). Moreover, reinforcing the progression of compression gradient by reducing tissue fluidity using Rac1 inhibitor significantly promoted the progression of epidermis stratification and follicle formation, indicated by the epidermal cell layer number and pattern geometry of follicle primordium (Figure 7d–i). Reducing tissue fluidity also increased the nuclear localization of *β*‐catenin protein, indicating the promotion of follicle cell fate determination (Figure S12, Supporting Information). On the contrary, increasing tissue fluidity by ROCK inhibitor attenuated the formation of follicle primordium (Figure S13, Supporting Information). In addition, disrupting the interfacial mechanical interaction between epidermal and dermal layers by inhibiting epidermal cell/base membrane adhesion using RGD largely attenuated the morphogenesis of primordium (Figure S14, Supporting Information). Thus, our findings suggest a model for the initiation of epidermis stratification during feather follicle formation that dermal cell aggregation induces mismatch deformation between epidermal layer and basement membrane, which generates a progressive compression gradient in epidermal layer and the latter triggers cell division reorientation leading to epidermis stratification.

## 3 Discussion

In this work, we propose a self‐organization mechanism for the initiation of morphogenesis during tissue growth, in which a progressive compression gradient caused by macroscopic interfacial mechanics drives the initiation of morphogenesis at certain developing stages in multilayered tissues. Our experiments and theoretical analysis show that mismatch deformation between adjacent layers during tissue growth induces progression of compression gradient by interfacial interactions, which triggers cell delamination and cell division reorientation in solid‐like state tissues and ultimately induces 3D deformation and tissue stratification. Meanwhile, in fluid‐like state tissues, the interaction between adjacent layers induced a dynamic progression gradient leading to 2D tissue deformation instead of 3D deformation (Figure 7j).

We also proposed the crucial role of progressive compression gradient in epidermis generated by the interaction between epidermal and dermal layers in the initiation of epidermal cell stratification during chicken skin development. Interestingly, in zebrafish embryos, the friction force between mesoderm and neurectoderm layers during cell moving has been reported to be a key determinant in the positioning of neural anlage.^[^ ^25^ ^]^ Thus, interfacial mechanical interaction between adjacent layers may be a conserved force origin during embryonic development.

Resembling to the instability process in film/substrate material system, we regard the cell sheet as a film, which is subjected to the interfacial shear stress during cell sheet expansion. Thus, the growing cell sheet will be compressed and buckled. We found that cell–cell adhesion, cell–substrate adhesion, and cell fluidity all affected the degree of cell sheet compression and tissue stratification. Interestingly, recent studies regard the process of cell aggregation and layering as the wetting problem and also reveal that inhibiting cell contraction, cell‐to‐cell adhesion, and cell‐to‐substrate adhesion have significant influences on the dewetting process.^[^ ^14^, ^26^ ^]^

Morphogenetic processes always occur at certain developmental stages during tissue growth. In *Drosophila* wing disk development, different cell proliferation rates induce mechanical strain to shape tissues and control tissue size by regulating proliferation and division orientation.^[^ ^27^ ^]^ Moreover, the constriction from the basement membrane is necessary for correct fold positions initiated by planar differential growth rates.^[^ ^28^ ^]^ In our experimental model, 2D and 3D morphogenetic processes emerge at the critical tissue size, which is determined by the critical value of the compressive strain generated by interfacial interaction during tissue growth. This macroscopic mechanical dissipation provides a possible regulatory mechanism for the spatiotemporal control of morphogenesis and size control of growing tissues.

It has been recognized that crowding is a common phenomenon in physiological and pathological processes and regulates tissue homeostasis maintenance.^[^ ^29^ ^]^ A recent study reports that during the development of zebrafish heart, proliferation‐induced crowding leads to tension heterogeneity that drives cell stratification.^[^ ^30^ ^]^ According to our results, critical compressive strain has profound effects on cell behaviors, including cell delamination, cell division reorientation, and in‐plane cell rearrangement, which all contribute to tissue shaping, homeostasis maintenance, and size control. Moreover, in our model experiment, the compression gradient is autonomously generated by tissue growth and therefore can well simulate the in vivo crowding conditions with the controlled crowding levels.

As an important characteristic of tissues, fluidity (jamming/unjamming state) is determined by collective cell motion and dynamically altered during tissue growth.^[^ ^31^ ^]^ Recent studies suggest that tissue fluidity plays crucial roles in homeostasis maintenance and tumor invasion.^[^ ^19^, ^32^ ^]^ Saadaoui et al. reported that myosin contractility‐induced global tissue flow contributed to morphogenesis during avian gastrulation.^[^ ^33^ ^]^ In our work, we found that tissue fluidity could affect the local accumulation of compressive strain under interfacial interaction between adjacent layers, which finally regulates tissue deformation patterns (2D or 3D). These findings may provide insights into the regulatory mechanism underlying morphogenesis by tissue fluidity.

There is no doubt that mechanical forces play essential roles in the regulation of morphogenesis. The role of cell‐scale forces generated by cytoskeleton contraction and transmitted by cell adhesions has been intensively studied in regulating tissue morphogenesis.^[^ ^1c^ ^]^ However, the upstream regulation mechanism of these molecular machines, especially at the tissue scale, is rarely investigated. Our results reveal that progression of the compression gradient controlled by the tissue‐scale interfacial shear stress has propounding effects on local cell delamination, reorientation of division plane, and cell intercalation to initiate morphogenesis. Moreover, the interfacial mechanics could be stemmed from mismatch deformation during cell migration, aggregation, proliferation, etc. Thus, this scale‐spanning mechanical loop from the cell scale to the tissue scale and then returning to the cell scale may be a fundamental self‐organized mechanism during the morphogenetic process in growing multilayered tissues. Further study is needed to elucidate the molecular mechanotransduction mechanism underlying the regulation of these cell behaviors by the progression of the compression gradient under interfacial mechanical interactions.

Taken together, our findings unveil a self‐organized mechanism that drives the initiation of morphogenesis in growing multilayered tissues. These results also open up a new avenue that tissue‐scale forces regulate cell behaviors, which could in turn facilitate tissue self‐organization.

## 4 Experimental Section

### Cell Culture and Immunofluorescence

HeLa, HepG2, MDCK, MDA‐MB‐231, U2OS, B16F10, and MEFs were cultured in dulbecco's modified eagle medium (DMEM) medium (containing with 4.5 g L^−1^ glucose, l‐glutamine, and sodium pyruvate) supplemented with 10% fetal bovine serum (FBS; Life technologies, CA, USA), and 100 IU mg^−1^ penicillin–streptomycin (Life technologies, CA, USA), and 1% (v/v) non‐essential amino acids (NEAA; Life technologies, CA, USA). For the experimental treatment, monoclonal culture was performed for the same period of time. Isolation of primary MEFs from mouse embryos was performed according to the protocol written by Xu.^[^ ^34^ ^]^ For monoclonal cell culture, cells were seeded at 1:200 dilution after cell density reaching to 70–80% confluence. The pharmacological agents were added including blebbistatin (Sigma, 25 × 10^−6^ m), RGD (Abcam, 50 µg mL^−1^), E‐cadherin neutralizing antibody (Biolegend, 10 µg mL^−1^), NSC23766 (Abmole, 20 × 10^−6^ m), Y27632 (Abmole, 20 × 10^−6^ m) if applicable. Images were taken on the Nikon microscope. Immunofluorescence was performed with the following primary antibodies: vinculin (Abcam ab129002, 1:100), *β*‐tubulin (Abcam ab6046, 1:100), NuMA (Abclonal A0527, 1:100). Cells were grown at 37 °C in an incubator with 5% CO_2_. Cells grown on glass bottom dishes were fixed with 4% paraformaldehyde for 10 min at room temperature. The cells were incubated with 5% bovine albumin (BSA), in phosphate buffered saline (PBS) +0.1% Tween 20 for 2 h. Cells were incubated with primary antibodies at the optimal concentrations (according to the manufacturer's instructions) at 4 °C overnight. After washing, cells were incubated for 2 h with secondary antibodies: 488/568/633 immunoglobulin G (IgG; H+L) and/or Alexa Flour 568/647 phalloidin (Invitrogen) for 1 h. Cell nucleus were stained with 4′,6‐diamidino‐2‐phenylindole (DAPI, Invitrogen) for 10 min at room temperature. Confocal images were taken on the Leica microscope equipped with a 10×, 40×, or 63× objective. Experiments were replicated at least three times.

### Different Types of ECM

The working solution were added including fibronectin (Corning, 20 µg mL^−1^), gelatin (Millipore, 0.1%), type 1 collagen (Sigma, 200 µg mL^−1^) if applicable, on a Petri dish and incubated at 37 °C in a humidified atmosphere of 5% CO_2_ for 1 h. For ECM with different elasticity which collagen on 1 kPa or 40 kPa polyacrylamide (PA) gel, please refer to the protocol written by Tse and Engler for more details.^[^ ^35^ ^]^ Briefly, a mixture of acrylamide and bis‐acrylamide at a specified concentration was polymerized on a glass slide, and then the gel was coated with sulfosuccinimidyl‐6‐[4′‐azido‐2′‐nitrophenylamino] hexanoate (Sulfo‐SANPAH; Pierce). After UV irradiation for 10 min twice, the polyacrylamide sheet was washed twice and incubated overnight with a solution of type 1 collagen (0.2 mg mL^−1^) at 4 °C.

### Live Imaging

Live cell imaging was performed in a Leica microscope or HoloMonitor M4, enclosed in an incubator to maintain the samples at 37 °C and 5% of CO_2_ throughout the experiments. Images were acquired every 10 min with Leica software. Spindle‐rocking experiments were acquired every 3 min with the Leica microscope. HoloMonitor M4 is a quantitative phase‐imaging‐based cell analyzer utilizing the principle of digital holographic microscopy. Live cell imaging was performed in HoloMonitor M4, enclosed in an incubator to maintain the samples at 37 °C and 5% of CO_2_ throughout the experiments. Images were acquired every 10 min with HStudio 2.7.

### PIV Measurement

PIV analysis was conducted using a custom algorithm based on the MatPIV software package for MATLAB. A series of live cell images of HeLa and MDCK were used to calculate the velocity of the cells in the cell sheet. The mean velocity was subtracted from calculated velocity fields to avoid any drift‐related bias and to get the velocity fields of the cells (the net movement of the cell cluster for its drift was less than 10% and could be ignored). The heat maps of magnitude were also exported subtracting the mean velocity. From the exported text files, the overall cell speed, or rms velocity *v*_rms_ was measured and the average *v*_rms_ of each type of cell was calculated. The correlation algorithm was coded using MATLAB in the lab. Experiments were replicated at least three times.

### Image Processing, Segmentation, and Quantification

In cell data analysis of HoloMonitor M4, cell area, cell thickness, cell sheet area, and cell sheet thickness were analyzed by the software HStudio 2.7. HStudio 2.7 can automatically segment and extract physical parameters of the cell. For more details, please refer to the official manual.

For the area of cell clones and individual cells, cell boundary labeling and areas were determined manually in Fiji.

For the cell thickness, width, and division angle, the collected confocal tomography images were imported into Bitplane imaris for 3D reconstruction. After reconstruction, *XZ* and *YZ* profiles were randomly selected to measure thickness, width, and cell division angle manually. Data were measured on three samples, using three regions of the image.

For the thickness, width, and division angle in chicken embryo skin, the collected confocal tomography images were imported into Bitplane imaris. Cell thickness, length, and division angle were measured manually on software.

For the pattern geometry in chicken embryo skin, diameter and spacing were measured manually using Fiji.

For shape index,^[^ ^36^ ^]^ a semiautomatic segmentation pipeline was used to determine the cell boundary. The blurry boundaries of the cells were manually enhanced. After processing the image by binary Ostu, the finally image was produced by Median filtering. The parameters were extracted by image segmentation to obtain the area and perimeter.

For cell trajectory, the cell tracking image was produced by software Bitplane imaris.

For cell rearrangement analysis, cell boundary labeling and areas were determined manually in Fiji.^[^ ^37^ ^]^

### Strain Field Analysis

The area of each cell was calculated by Fiji software, and the compression strain of each cell was calculated according to Equation (S3) (Supporting Information). Finally, origin software was used to draw the compression strain diagram. The strain field was obtained by analyzing variation of cell area using Equation (S4) (Supporting Information).

### Finite Element Simulations

Finite element simulations were performed using commercial software ABAQUS (2016). Details of the finite element method are present in the Supporting Information. In the finite element model, the growing cell monolayer sheet was placed on a stiff substrate with the interfacial fraction factor being controlled. The cell sheet was under isotropic expansion to simulate cell proliferation and growth. The cell sheet was modeled as the linear elastic material. No other boundary conditions were applied on the cell sheet.

### Skin Culture and Immunofluorescence

Fertilized eggs were bought from local farms. The eggs were cultured in a moist environment at 37 °C and staged according to Hamburger and Hamilton.^[^ ^38^ ^]^ Dorsal skin pieces were dissected from E6 embryos and spread flat on the polycarbonate membrane (pore size: 0.4 µm, Corning, Cat. No.3413). Please refer to the protocol written by Chuong for more details.^[^ ^39^ ^]^ Culture was DMEM with 2% chick serum and 10% FBS along with pharmacological agents NSC23766 (Abmole, 20 × 10^−6^ m), RGD (Abcam, 100 µg mL^−1^) if applicable. Skin pieces were cultured for 48 h at 37 °C before being fixed in 4% paraformaldehyde in PBS. Images were taken on the Nikon stereoscopic microscope. Pattern geometry was calculated using Fiji to measure the spacing and diameter. Immunofluorescence was performed with the following primary antibodies: *β*‐tubulin (Abcam ab6046, 1:100), *β*‐catenin (Abcam ab16051, 1:100). For immunofluorescence staining, embryos or cultured dissected skin pieces were fixed in 4% paraformaldehyde in PBS and embedded in optimal cutting temperature compound (OCT; SAKURA, ref.4583). The tissue blocks were placed in liquid nitrogen for 1 min and in frozen section. The section thickness was about 14 µm. The sections were incubated with 5% BSA, in PBST (PBS + 0.1% Tween 20) for 2 h. The sections were incubated with primary antibodies at the optimal concentrations (according to the manufacturer's instructions) at 4 °C overnight. After being washed with PBS, the sections were incubated for 2 h with secondary antibodies: 488/568/633 IgG(H+L) for 1 h. Nuclei were stained with DAPI (Invitrogen) for 10 min at room temperature. Confocal images were taken on the Leica microscope equipped with a 10×, 40×, or 63× objective. Experiments were replicated at least three times. All the treatments were carried according to regulations for the Administration of Affairs Concerning Experimental Animals promulgated by Decree No.2 of the State Science and Technology Commission of China and the Guiding Principles for the Care and Use of Animals set by Beijing Government. All protocols were approved by the Animal Care Committee of Beihang University.

### Quantification and Statistical Analysis

Data were presented as mean ± standard error of mean (SEM), and boxplot format data were presented as median ± min/max, as indicated in the corresponding figure legends. Sample size (*n*) for each statistical analysis was presented as box plots of all quantifications, and was indicated in the figure legends. Statistical significance was determined by the two‐tailed Student's *t*‐test and one‐way ANOVA with Tukey's correction, as indicated in the corresponding figure legends. Tukey's posthoc test was used for multiple posthoc comparisons to determine the significance between the groups after one‐way analysis of variance (ANOVA). *p* < 0.05 was considered statistically significant. Statistical analysis was carried out using commercial software IBM SPSS Statistics 22.

## Conflict of Interest

The authors declare no conflict of interest.

## Author Contributions

T.L.C. and Y.Z. contributed equally to this work. T.L.C., Y.Z., J.D., Y.P.C., and Y.B.F. designed the study, performed, and interpreted experiments. X.B.Z. and S.K.L. helped with cell experiments and statistical analysis. J.L.C. helped with PIV analysis. Y.Z. and Y.P.C. did mechanical analysis. J.D., Y.P.C., and Y.B.F. conceived and supervised this project and prepared the paper.

## Supporting information

Supporting Information

Supplemental Movie 1

Supplemental Movie 2

Supplemental Movie 3

Supplemental Movie 4

Supplemental Movie 5

## Data Availability

The data that support the findings of this study are available from the corresponding author upon reasonable request.
