# Supplementary material for: Self‐Organization of Tissue Growth by Interfacial Mechanical Interactions in Multilayered Systems
Source: Adv Sci (Weinh). 2022 Feb 9;9(13):2104301. doi: 10.1002/advs.202104301 (PMC9069393; doi:10.1002/advs.202104301)
Supplement: Supplementary file 1 — Supporting Information [file ADVS-9-2104301-s005.pdf]

ADVANCED SCIENCE
Open Access

Supporting Information

for *Adv. Sci.*, DOI 10.1002/advs.202104301

Self-Organization of Tissue Growth by Interfacial Mechanical Interactions in Multilayered Systems

Tailin Chen, Yan Zhao, Xinbin Zhao, Shukai Li, Jialing Cao, Jun Guo, Wanjuan Bu, Hucheng Zhao, Jing Du, Yanping Cao* and Yubo Fan**

ADVANCED SCIENCE
Open Access

Supporting Information

for *Adv. Sci.,* DOI: 10.1002/advs.202104301

Self-Organization of Tissue Growth by Interfacial Mechanical Interactions in Multilayered Systems

Tailin Chen, Yan Zhao, Xinbin Zhao, Shukai Li, Jialing Cao, Jun Guo, Wanjuan Bu, Hucheng Zhao, Jing Du, Yanping Cao,* and Yubo Fan**

Supplementary Materials

Theory model

The theoretical model is shown in Fig. 2a. The cell monolayer is modeled as a thin film, which is attached on the surface of a rigid substrate. Direction of the tangential adhesion force between the cell and substrate is contrary to the relative motion. Thus, compression would be induced when the cell monolayer is growing on the substrate. The tangential adhesion force is assumed as $f = f(r)$, where r is the distance between the cell and the center of the monolayer. f is concerned with the tangential adhesion between the cell and substrate, which is related to the cell type, stiffness of substrate and the rate of cell division or growth. By analyzing the stress state of the monolayer and solving the equilibrium equation, one can obtain the distribution of the equi-biaxial compressive stress in the monolayer

$$\sigma_r = \frac{1}{h}\int_r^R f \, \mathrm{d}r \tag{1}$$

Here R is the outer radius of the cell monolayer, and h is the thickness of cell sheet. The compressive strain can be obtained by the constitutive relation as

$$\varepsilon_r = \frac{1-\nu}{Eh}\sigma_r \tag{2}$$

where E, ν are the modulus and Poisson's ratio, respectively. The central region of the monolayer would sustain higher level of compression than the cells in other regions. Thus, cell extrusion is most likely to occur in the central region, which is consistent with the experimental observations. The maximum compressive strain in the cells can

be obtained as

$$\varepsilon_{\max} = \frac{1-\nu}{Eh}\int_0^R f \, \mathrm{d}r \tag{3}$$

which depends on the cell-substrate interactions and area of the cell monolayer. Based on Eq. (2), one can also obtain the cell area

$$A/A_0 = (1-\varepsilon)^2 \tag{4}$$

where A_0 is the normal area of a full-grown cell without sustaining compression. Eq. (4) demonstrates that distribution of cell area can reflect the strain state in the cell monolayer. With cell proliferation, radius of the cell monolayer increases, and area of cells in the central region is reduced, indicating that the cell monolayer has a high level of compression in the central region.

Given the distribution of the interfacial adhesion force, one can obtain the distribution of the compressive strain and cell area of the cell monolayer. In this work, the tangential adhesion force is assumed to be uniform over the cell monolayer. Thus, the maximum compressive strain in the cell and the distribution of the cell area can be given by

$$\varepsilon_{\max} = \frac{1-\nu}{Eh} fR \tag{5}$$

$$A/A_0 = \left[1 - \frac{1-\nu}{Eh} f(r-R)\right]^2 \tag{6}$$

Based on Eq. (4), distribution of the compressive strains in the HeLa cells in experiments can be obtained by analyzing the distribution of cell area, which is shown in Fig. 1b. The sequence in Fig. 1b shows the morphologies of growing HeLa cells with the distribution of the compressive strains given in Fig. 4. The size of the cell monolayer sheet grows bigger along with the cell proliferation, generating higher level of

compressive stress in the monolayer. Areas of individual cells in the central region are reduced due to the increased compressive strains. The experimental observations and calculations are consistent with the theoretical predictions.

To validate the theoretical model, finite element simulations are performed to explore the relation between the cell-substrate interactions and stress field in growing cells. Results of finite element simulations are shown in Fig. 2d. In the finite element model, the growing cell monolayer sheet was placed on a stiff substrate with the interfacial fraction factor being controlled. The cell sheet was under isotropic expansion to simulate the growth. More than 12,000 linear hexahedral elements were adopted to in the simulations. The cell sheet was modeled as the linear elastic material. With the increase of the growth strain, compressive stresses in the cell monolayer are generated due to the cell-substrate interactions, and the central region has a higher level of compressive stress. The stress level can be reduced by regulating the cell-substrate interactions (Fig. 2d). Finite element simulations are consistent with experimental observation and theoretical analysis.

The elastic strain energy stored in the cell would also increase with the cell proliferation. When the elastic strain energy in the cell is small, interfacial normal adhesion would impose restriction on cell extrusion, making the cell monolayer grow in plane. Thus, higher compressive stress would be generated further. When the compressive stress reaches a critical value, elastic strain energy stored in the cell may be greater than the energy needed for the occurrence of the interfacial delamination. In this critical condition, cell extrusion may happen, and the elastic strain energy can be

released. The critical condition for the cell extrusion can be written as

$$\gamma A = \frac{E}{1-\nu}\varepsilon_{\max}^{2}V \tag{7}$$

The left hand and right hand of the equation refer to the energy for the interfacial delamination and elastic strain energy in the cell, respectively. γ is the energy per area for the interfacial delamination, which is related to the interfacial normal adhesion between the cell and substrate. $\varepsilon_{\max}$ is the maximum strain in the cell, and V is the cell volume. Eq. (6) demonstrates that there exists a critical area or size of the cell monolayer at the critical condition of cell extrusion. The critical size of the cell monolayer sheet depends on the mechanical properties of cell and cell-substrate interactions. The cell extrusion can be controlled by regulating the modulus of the cell and cell-substrate interactions.

When the normal adhesion between the cell monolayer and the substrate is enhanced, cell extrusion would be more difficult to happen, and the cell would bear larger compression before extrusion. When the tangential adhesion is larger, higher level of compression would be induced, and the cell extrusion is more likely to happen. The adhesion between the cells may have effect on the cell sheet stress. Maximum compressive stress in the cell monolayer may be reduced when lowering down the adhesion between the cells. Thus, cell extrusion would be more difficult to happen. Cytoskeleton is the main components that determine the cell mechanics. The cytoskeleton can bear compressive stress and store elastic strain energy. If the cytoskeleton in the cell is suppressed, cell extrusion is hard to happen. These predictions were confirmed by experiments (Fig. 2e-g).

Shape of the cell can be changed due to the compression induced by the interfacial shear stress. Thus, the orientation of the cell division may be altered. During the evolution of the cell monolayer sheet, cell shape in the central region was changed from flat to columnar due to the high level of compression stress. While in the periphery of the monolayer, cell shape maintained flat. Stretch ratio of the cell in the direction of thickness can be written as

$$\lambda = 1 + \frac{2\nu}{1-\nu}\varepsilon_r \tag{8}$$

Base on the stretch ratio in Eq. (8), cell thickness/width characterizing cell shape can be obtained as

$$\frac{h}{b} = \frac{\lambda}{1-\varepsilon_r}\frac{h_0}{b_0} \tag{9}$$

Here h and b refer to the cell thickness and width. h_0 and b_0 refer to the initial thickness and width of the cell without sustaining compression. In the periphery of the monolayer, cell thickness/width is small since the compressive strain in the cell is small. While in the central region with high level of compressive strain, cell thickness/width can be very large. This phenomenon of cell shape variation induced by the interfacial mechanical interaction is also confirmed by the finite element simulations, as shown in Fig. 3c.

Supplementary Figures

Supplementary Figure S1

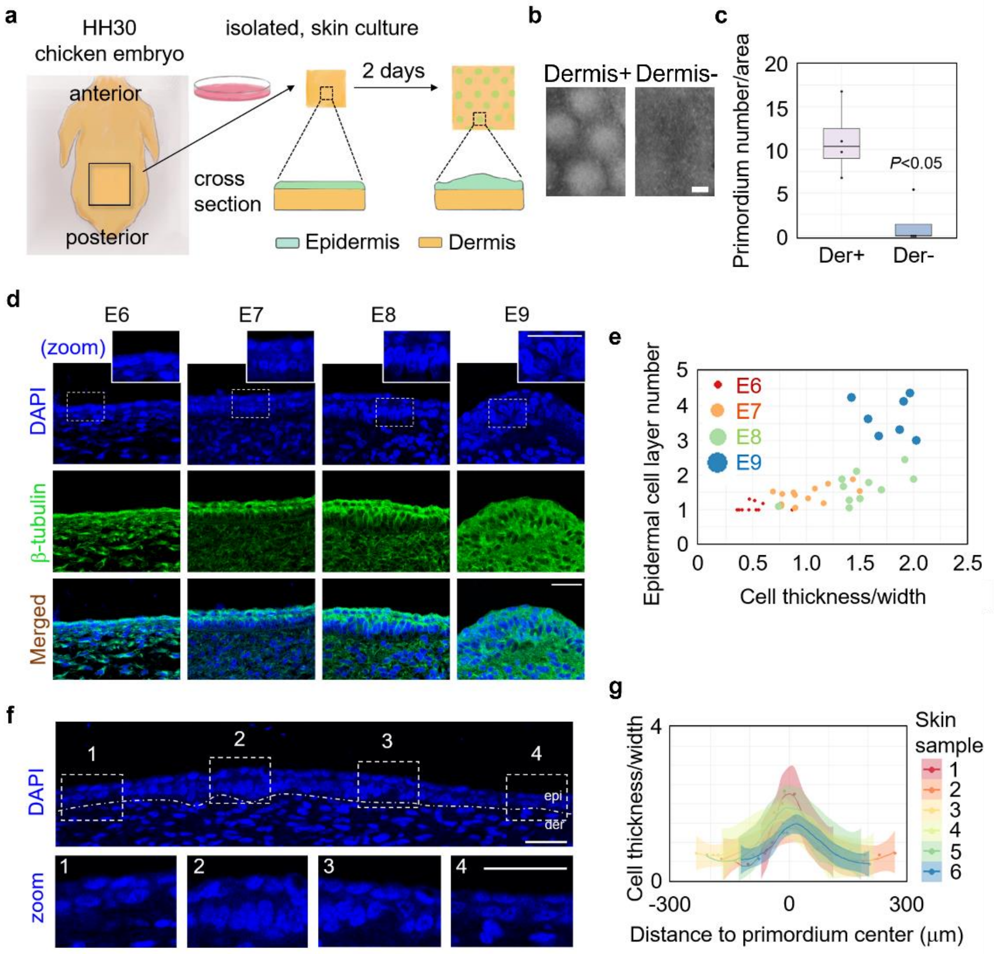

Figure S1. Progressive compression gradient is strengthened in epidermal layer during chicken feather follicle morphogenesis. (a) The illustration of experimental workflow for studying the epidermal morphogenesis in cultured chicken skins. (b) Presentative bright field images of ex vivo culture of embryonic chicken epidermis with (Der+) or without (Der-) dermal cell layer. No primordium was formed in isolated epidermis cultured without dermal cell layer. Scale bar: 500 μm. (c) The statistical

analysis of primordium number per area (9 mm^2) in (b) ($n = 4$ embryos). Data are presented as median ± min/max. Two-tailed independent-samples t test. (d) Images of skin tissues from different stages of chicken embryos showing the deformation of epidermal cell shape. Scale bar: 25 μm. (e) The statistical analysis of cell deformation (thickness/width) and cell layer number of epidermis in skin tissues from different stages of chicken embryos. Each dot represents the average value of an embryo. Three primordia were selected from each embryo for statistics. E6 ($n = 10$ embryos), E7 ($n = 12$ embryos), E8 ($n = 11$ embryos), E9 ($n = 7$ embryos). (f) Images of epidermal cell shape deformation at different location around the center of primordium. Scale bar: 25 μm. (g) The statistical analysis of cell deformation (thickness/width) with different distance to the center of primordium. $n = 6$ embryos.

Supplementary Figure S2

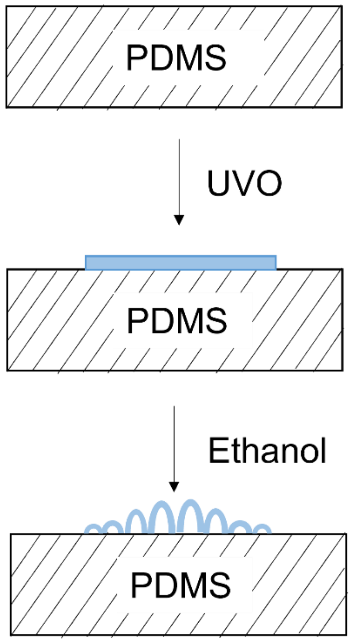

Figure S2. Surface wrinkling induced by differential expansion in a film-substrate system[11b]. Polydimethylsiloxane (PDMS) was exposed to UV/Ozone (UVO) for 10 – 55 minutes to form a stiff solvent-responsive oxide layer (highlighted in blue) on the surface. Then dropping an ethanol/glycerol mixture solution containing 60% – 100% ethanol by volume to induce surface wrinkling.

Supplementary Figure S3

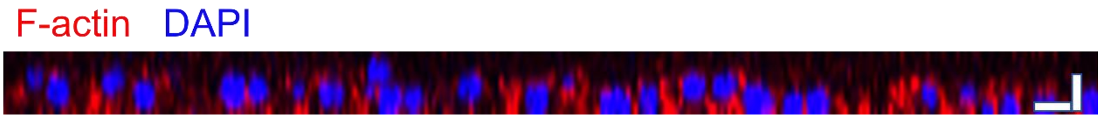

Figure S3. Representative XZ section images of B16F10 cell sheet. F-actin and nucleus were stained by Phalloidin and DAPI respectively in B16F10 cell sheet. Scale bar: 15 μm.

Supplementary Figure S4

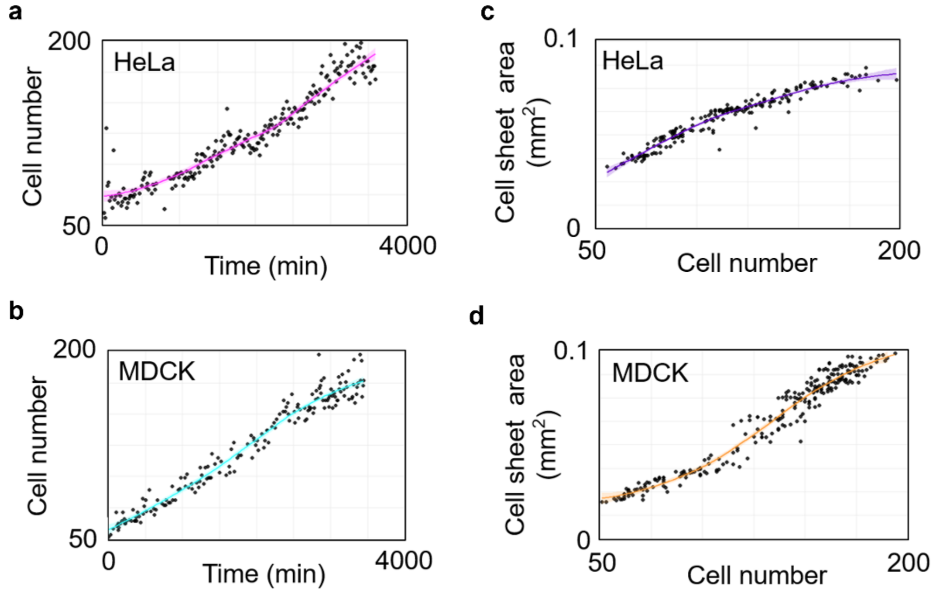

Figure S4. Proliferation and expansion of cell sheets during growth. The statistical analysis of the cell number during HeLa cell sheet (a) and MDCK cell sheet (b) growth. The statistical analysis of the cell sheet area during HeLa cell sheet (c) and MDCK cell sheet (d) growth.

Supplementary Figure S5

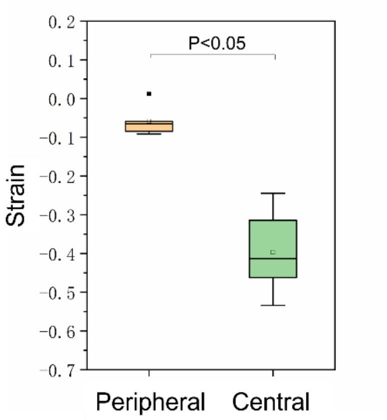

Figure S5. Single cell tracing of compressive strain. The plane strain of single cells in central and peripheral regions of cell sheets during 24 hours analyzed by holographic imaging cytometer (n = 6). Data are presented as median ± min/max. Two-tailed independent-samples t test.

Supplementary Figure S6

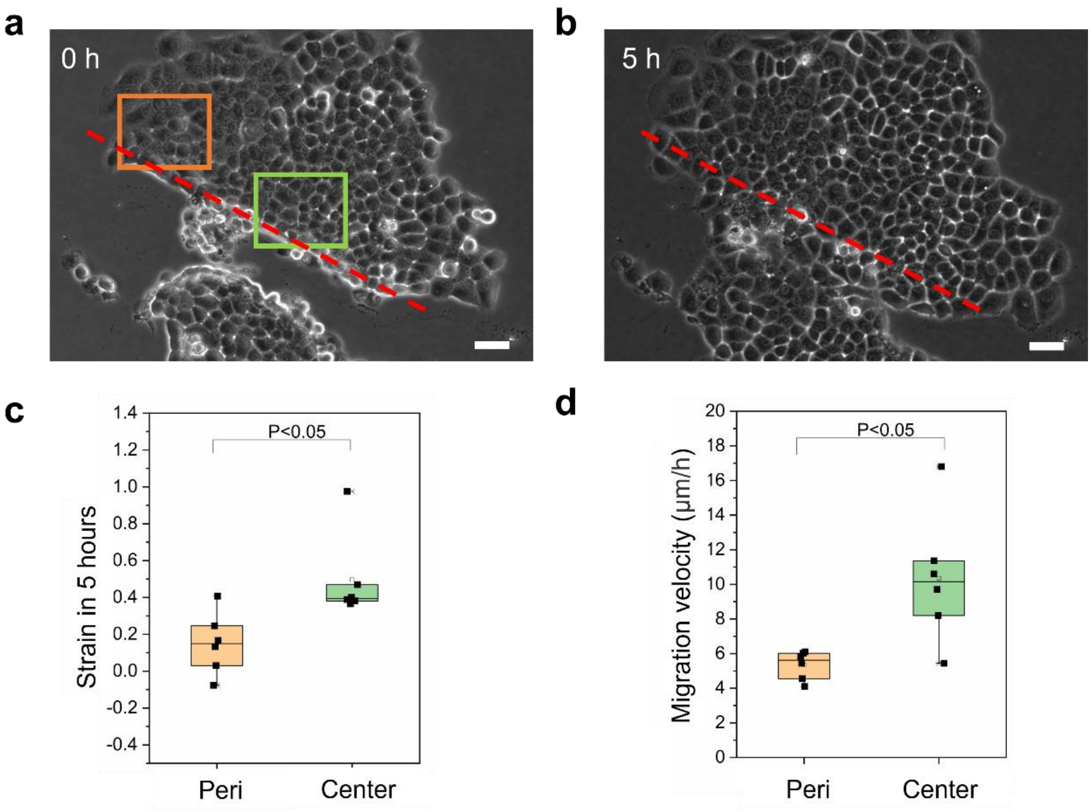

Figure S6. Cell sheet scratch assay. The representative image of cell sheet upon (a) and 5 hours after (b) scratch passing through the center (green box) and edge (orange box) of the cell sheet. The expansion (c) and migration speed (d) were compared between cells in the central region and peripheral region of the cell sheet. Scale bar: 50 μm, (n = 6). c, d) Data are presented as median ± min/max. Two-tailed independent-samples t test.

Supplementary Figure S7

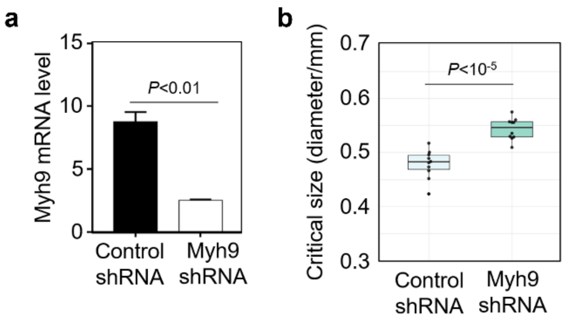

Figure S7. Inhibition of Myosin effects the emergence of 3D morphogenesis. (a) The mRNA level of myosin heavy chain 9 gene (myh9) in HeLa cells transfected with myh9 shRNA and control shRNA. (n = 3). Data are presented as mean ± SEM. (b) The critical sizes for morphogenesis in myh9 shRNA cells and control cells. (n = 10). Data are presented as median ± min/max. a, b) two-tailed independent-samples t test.

Supplementary Figure S8

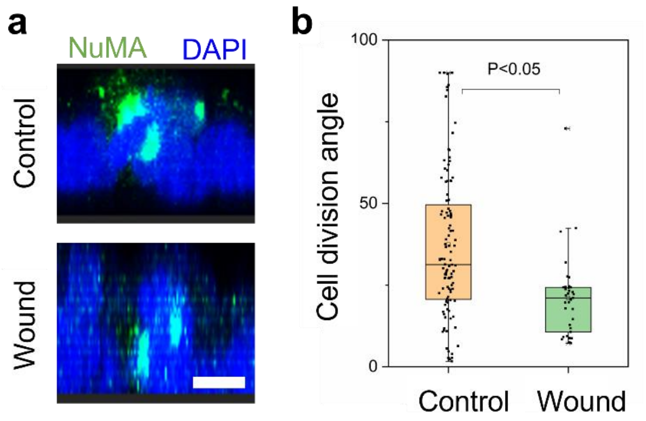

Figure S8. Cell division angle after scratch. (a) Representative XZ slice image of NuMA and nucleus stained by NuMA antibody and DAPI respectively in the control and wound of HeLa cell sheet. (b) The cell division angle in the center of cell sheet without (Control) or with (Wound) scratch. Scale bar: 10 μm. (Control: n = 116 cells from 18 clones. Wound: n = 35 cells from 9 clones). Data are presented as median ± min/max. Two-tailed independent-samples t test.

Supplementary Figure S9

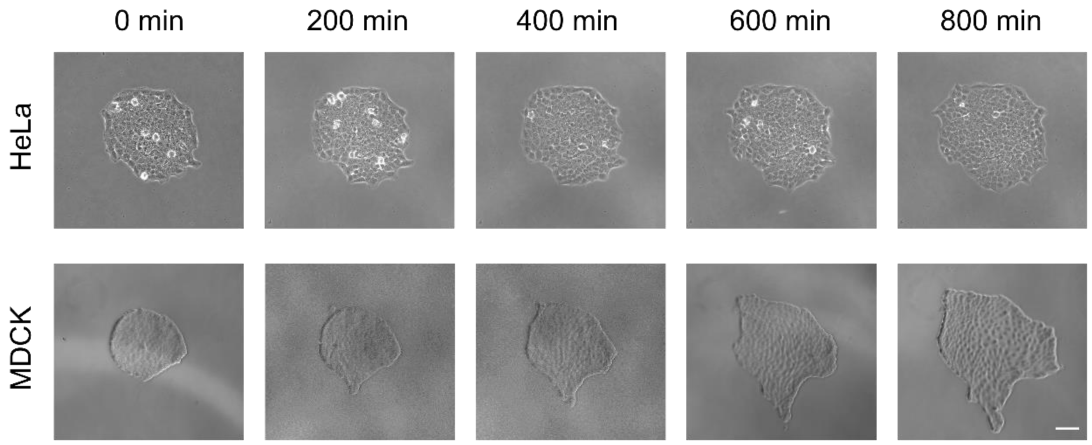

Figure S9. Different deformation pattern between HeLa and MDCK cell sheets during growth. Representative phase contrast images of a growing monoclonal HeLa and MDCK cell sheet captured at the indicated time (min: minute). Scale bar: 100 μm.

Supplementary Figure S10

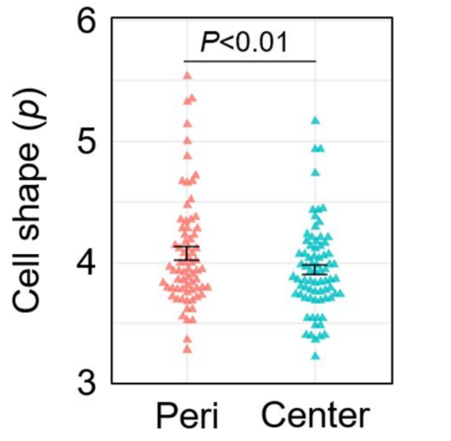

Figure S10. Cell shape index in the peripheral and central regions of HeLa cell sheet. The statistical analysis of cell shape index of cells in the peripheral region and central region of HeLa cell sheet. (n = 75). Data are presented as mean ± SEM. Two-tailed independent-samples t test.

Supplementary Figure S11

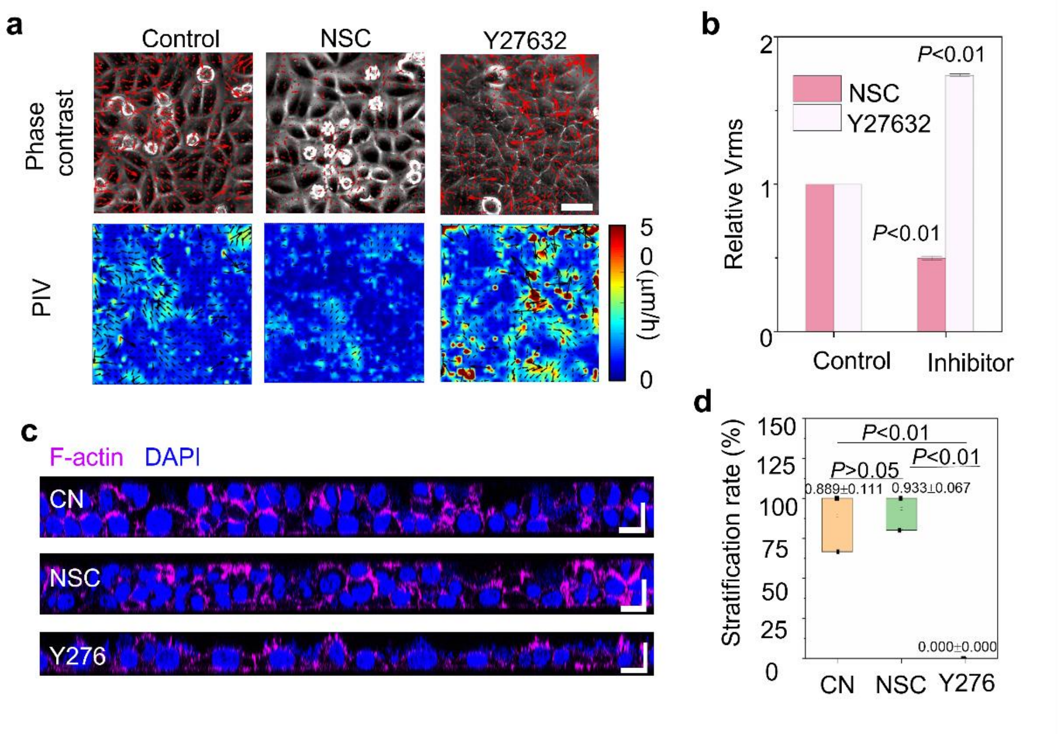

Figure S11. Tissue fluidity effects on Hela cell sheet stratification. (a) The velocity field superimposed on the corresponding phase contrast (upper panel) and velocity map (lower panel) images measured by PIV of Hela cell sheet in the presence of inhibitors or vehicle. Scale bar: 20 μm. (b) The statistical analysis of cell speed (rms velocity) measured by PIV of HeLa cell sheets in the presence of inhibitors or vehicle. (NSC: n = 6, Y276: n = 7). Data are presented as mean ± SEM; Two-tailed one-sample t test. (c) Representative XZ section images of Hela cell sheet in the presence of inhibitors or vehicle. Scale bar: 25 μm. (d) The statistical analysis of stratification rate of Hela cell sheet in the presence of inhibitors or vehicle. Stratification rate values (mean ± SEM) are indicated above the graph. (CN: n = 6, NSC: n = 6, Y276: n = 7). Data are presented as median ± min/max; One-way ANOVA with Tukey's correction.

Supplementary Figure S12

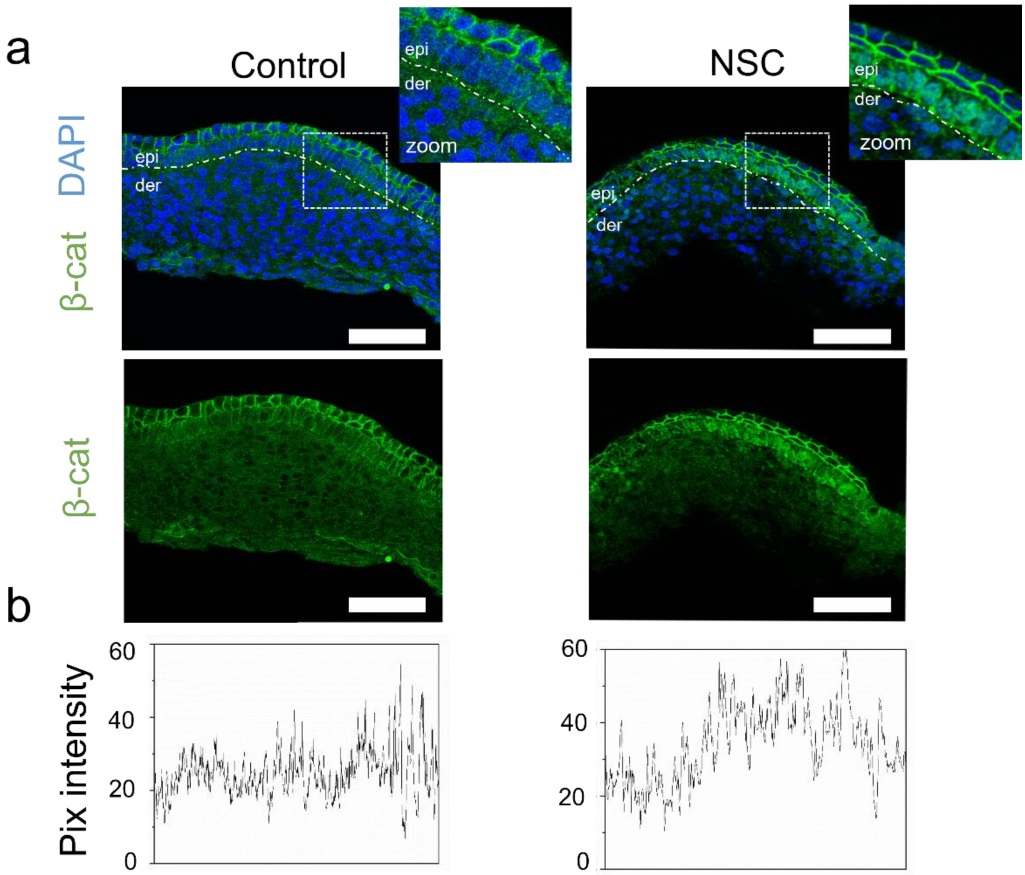

Figure S12. Nuclear localization of β-catenin in chicken epidermal cells regulated by Rac1 inhibitor. (a) Cross-section of embryonic chicken skin stained with DAPI and β-catenin antibody. White dotted line separates epidermis (epi) from dermis (der). The enlarged image refers to the position of the white dotted box. (b) β-catenin fluorescence intensity was quantified by using the Fiji line (7 μm width) plot profile function to analyze a free-hand line drawn through the contour of the epidermis. Scale bar: 50 μm.

Supplementary Figure S13

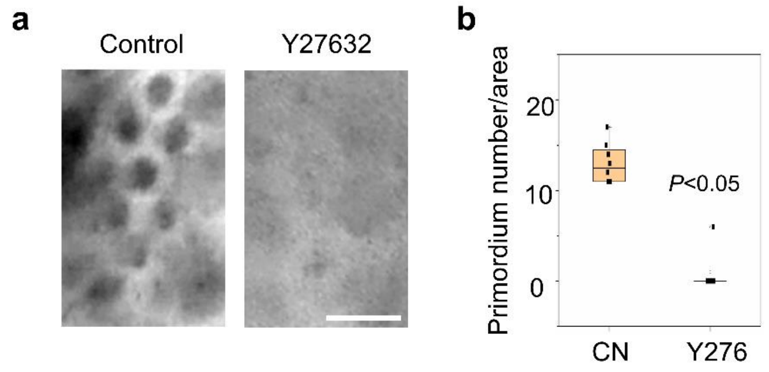

Figure S13. ROCK inhibitor suppressed the morphogenesis of primordium. (a) Ex vivo culture of embryonic chicken skin with or without Y27632. Scale bar: 1 mm. (b) The statistical analysis of primordium number per area (9 mm^2) (CN: n = 8, Y276: n = 7). Data are presented as median ± min/max. Two-tailed independent-samples t test.

Supplementary Figure S14

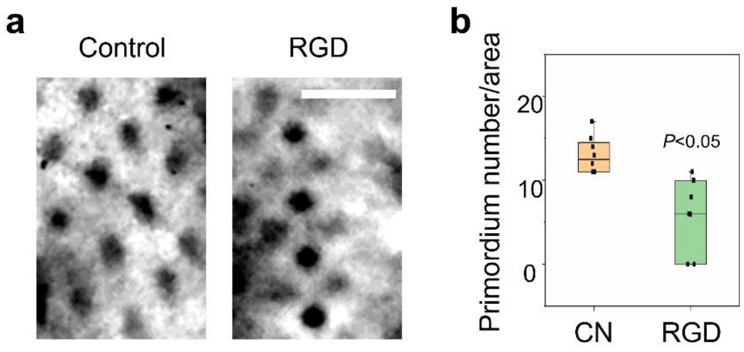

Figure S14. Disrupting the interfacial mechanical interaction between epidermal and dermal layers attenuated the morphogenesis of primordium. (a) Ex vivo culture of embryonic chicken skin with or without RGD. Scale bar: 1 mm. (b) The statistical analysis of primordium number per area (9 mm^2) (CN: n = 8, RGD: n = 7). Data are presented as median ± min/max. Two-tailed independent-samples t test.

Supplementary Table

ECM type	Critical diameter(μm)	N
No pre-coating ECM	463.8±18.9	10
Fibronectin	493.8±23.9	10
Gelatin	489.1±19.8	10
Collagen	520.3±21.8	10
Collagen (40 kPa PA gel)	413.8±9.6	5
Collagen (1 kPa PA gel)	342.6±26.0	4

Table S1. The critical size (mean ± SEM) for 3D morphogenesis of cell sheets growing on different types of ECM (No pre-coating ECM, Fibronectin, Gelatin, Collagen) and substrate with different stiffness made by polyacrylamide (PA) gel with elastic moduli of 40 kPa or 1 kPa.

Supplementary Movies

Supplementary Movie 1. Symmetric cell division before critical compression during HeLa cell sheet growth visualized by HoloMonitor M4 time-lapse cytometer.

Supplementary Movie 2. Asymmetric cell division after critical compression during HeLa cell sheet growth visualized by HoloMonitor M4 time-lapse cytometer.

Supplementary Movie 3. PIV analysis during HeLa and MDCK cell sheets growth.

Supplementary Movie 4. Time-lapse imaging of MDCK cell sheet during growth.

Supplementary Movie 5. PIV analysis during MDCK cell sheet growth in the presence of inhibitors or vehicle (control).
